# Postoperative Biochemical Outcomes in Metabolic Bariatric Surgery: Results from a High-Adherence Cohort

**DOI:** 10.3390/jpm15010007

**Published:** 2024-12-27

**Authors:** Maria Sofia, Marcello Agosta, Sara D’Amato, Giuseppe Nicolò Conti, Chiara Mazzone, Gloria Faletra, Gaetano La Greca, Saverio Latteri

**Affiliations:** 1 Department of General Surgery, Cannizzaro Hospital, 95126 Catania, Italy; mariasofia2002@libero.it (M.S.); glagreca@unict.it (G.L.G.); saverio.latteri@unict.it (S.L.); 2 Department of Medical, Surgical Sciences and Advanced Technologies “G.F. Ingrassia”, University of Catania, 95123 Catania, Italy; chiaramazzone1995@gmail.com (C.M.); gloria.faletra@gmail.com (G.F.); 3 Department of Biomedical and Biotechnological Sciences, University of Catania, 95123 Catania, Italy; damatosara1998@gmail.com (S.D.); giuseppecontichimica@gmail.com (G.N.C.)

**Keywords:** bariatric surgery, obesity, follow-up, diabetes, weight loss, adherence to therapy

## Abstract

Background/Objectives: In metabolic bariatric surgery, structured follow-up protocols may play an essential role in achieving optimal patient outcomes. This study aims to report postoperative biochemical outcomes in a cohort of post-bariatric patients who underwent a structured follow-up protocol. Methods: This retrospective study included patients who underwent metabolic bariatric surgery and completed a one-year follow-up at Cannizaro Hospital from October 2022 to May 2024. Anthropometric, clinical, and laboratory data were collected for each patient at five different timepoints: baseline, 1, 3, 6, and 12 months post-surgery. All data were organized into a database and analyzed through descriptive statistics. Results: The study cohort (*n* = 80) showed a follow-up adherence equal to 97.5%. The mean value of BMI decreased from 42 to 27 one year after surgery. Lipid profiles improved, with significant reduction in total cholesterol and triglycerides and increase in HDL levels; LDL levels decreased initially but at one year returned to baseline. Thyroid hormones TSH and fT3 decreased significantly, while fT4 remained stable. A reduction in hepatic inflammation was observed, as evidenced by the decrease in GGT and transaminase levels. Pancreatic enzymes showed an initial increase but stabilized at the subsequent timepoints. Glycemic control improved, with statistically significant reductions in insulin, HbA1c, and glucose levels, and complete remission of type 2 diabetes mellitus in affected patients. Conclusions: This study shows significant weight loss and improved health outcomes in post-bariatric patients who underwent a structured follow-up protocol, suggesting the potential benefits of metabolic bariatric surgery.

## 1. Introduction

Obesity is a complex, multifactorial disease that results from the interaction of genetic, environmental, and behavioral factors, posing significant risks for chronic conditions like cardiovascular diseases, type 2 diabetes mellitus, and certain cancers [1,2]. Metabolic bariatric surgery (MBS) is a valid option for patients who failed to lose weight or to maintain long-term weight loss despite appropriate non-surgical treatment.

Post-bariatric follow-up has always been the Achilles heel of metabolic bariatric surgery [3]. Guidelines suggest close follow-up, where the patient must be followed by a multidisciplinary team to monitor complications, modify the therapeutic plan, and update the dietary plan [4]. The reality is that loss to follow-up is a major concern in MBS [5,6,7]. Only 40% of MBS studies meet the minimum criteria for adequate follow-up, which should be at least 80% of the original patient cohort [7]. The possible reasons behind loss to post-bariatric follow-up are several and multifactorial, ranging from inaccurate treatment to socio-psychological factors.

Experiences of poor and inaccurate treatment, such as brief and generalized follow-up, limited educational support, and lack of personalized treatment plans, may cause mistrust of health services, avoidance of care, and a decrease in treatment adherence rates [8]. Barriers to adherence and successful maintenance of weight loss could also include lack of support and motivation [9,10]. Lack of monitoring by healthcare professionals can result in feelings of abandonment and a drop in motivation [11]. Gaps in nutritional knowledge and lack of awareness about post-bariatric complications constitute additional factors contributing to poor adherence to therapy [9,10]. The result is worrying: one year after surgery, adherence can be as low as 30% [3].

The aim of this study is to report postoperative biochemical outcomes following metabolic bariatric surgery (MBS) in a cohort of patients who have undergone a structured follow-up protocol, thereby contributing to the ongoing discussion regarding the impact of follow-up on patient results.

## 2. Materials and Methods

### 2.1. Study Group

This retrospective study exclusively analyzed pre-existing, anonymized clinical data without any patient interaction or experimental intervention. All patients, however, had provided informed consent at the time of clinical management for the use of their anonymized data in research. Ethical approval was not required for this study in accordance with Article 110-bis of Legislative Decree 196/2003, as amended by Legislative Decree 101/2018, which allows the use of anonymized data for research purposes.

This retrospective, observational study included a cohort of patients who underwent metabolic bariatric surgery and completed follow-up at Cannizzaro Hospital (Catania, Italy) from October 2022 to May 2024. Patients were included in the study based on the following criteria: (1) indication for MBS; (2) body mass index (BMI) > 35; (3) age between 18 and 65 years; (4) failure to achieve significant weight loss despite numerous diets attempts and pharmacological therapy; (5) one-year post-bariatric follow-up.

Demographic and anamnestic data were collected through a structured interview conducted at the time of patient enrolment.

For each patient the following data were collected: age; gender; BMI; dietary history; smoking; obesity-related diseases such as type 2 diabetes mellitus, cardiovascular diseases, endocrine disorders, and history of malignancies; preoperative hematological assessment including evaluation of liver, renal, thyroid, and glycometabolic functions.

### 2.2. Operative Procedures

**Figure 1:**
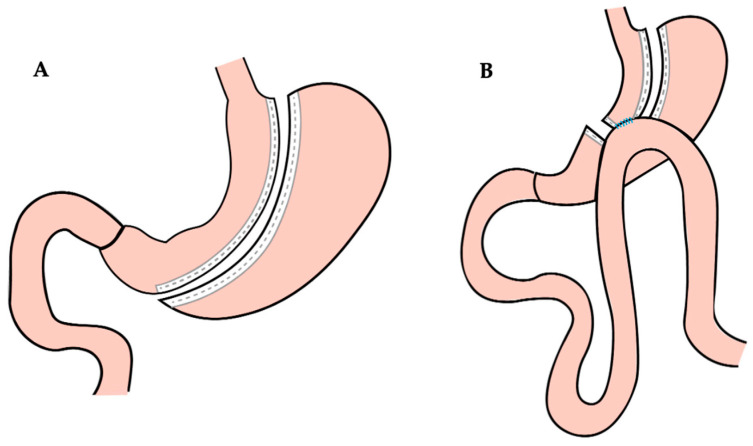
(**A**) Sleeve gastrectomy procedure; (**B**) one-anastomosis gastric bypass procedure.

All patients were operated under general anesthesia and were given perioperative antibiotics according to hospital protocol. The surgical interventions included sleeve gastrectomy (SG) and one-anastomosis gastric bypass (OAGB) (Figure 1). SG involves the removal of a large portion of the stomach, leaving a sleeve-shaped stomach. The SG is performed resecting the stomach along a 36-French bougie, starting 5 cm from the pylorus and extending to the area adjacent to the cardia. OAGB consists of creating a small stomach pouch and bypassing a portion of the small intestine, which reduces the absorption of calories and nutrients. The gastric pouch was constructed with a 36-French bougie, with the section made at the level of the gastric lesser curvature notch. A 180 cm loop was created from the Treitz ligament. The omega-shaped gastro-jejunal anastomosis was created using a powered stapler, and the entry site was closed with a continuous suture.

### 2.3. Follow-Up Data Collection

Medical records such as anthropometric, clinical, and laboratory data were collected for each patient at five different timepoints: baseline, 1, 3, 6, and 12 months post-surgery. During each follow-up visit, the following procedures were performed:

- Interview to assess patients’ dietary habits, quality of life, potential postoperative complications, and pharmacological therapy modifications;
- Collection of blood samples and evaluation of hematological parameters including evaluation of liver, renal, thyroid, and glycometabolic functions.

The outcomes of bariatric surgery, including weight loss, BMI value, % of total weight loss (%TWL), and changes in obesity-related diseases, were reported according to standard outcome criteria from the American Society for Metabolic and Bariatric Surgery [12].

All data were organized into a database and then analyzed through descriptive statistics.

### 2.4. Statistical Analysis

The analysis of clinical parameters was performed through descriptive statistics. Shapiro–Wilk and Kolmogorov–Smirnov normality tests were carried out to assess the normality of data distribution. A repeated-measures one-way ANOVA test for paired samples, with multiple comparisons, was used to analyze the changes across timepoints as regards normally distributed data. For parameters that did not show a normal distribution, the Friedman test followed by Dunn’s multiple comparisons test was performed to analyze the differences between timepoints.

Pearson’s and Spearman’s correlation coefficients were obtained to determine the correlation between quantitative variables. The obtained results were considered significant for a *p* < 0.05. All the statistical tests were performed using GraphPad Prism version 9.5.1 for MacOS, GraphPad Software, Boston, MA, USA, www.graphpad.com.

## 3. Results

### 3.1. Study Group

The study included 80 patients, of whom 82.5% were female (66) and 17.5% were male (14). The median age was 40 years (range 30–51) with the patients almost equally distributed among quartiles. Of the 80 patients included, 83.75% underwent SG (67) and 16.25% (13) underwent OAGB surgery. During the postoperative follow-up period, no complications were observed except in one patient who died on postoperative day seven due to sepsis following a gastric leak.

Table 1 shows the rate of comorbidities reported by patients before surgery. The six patients affected by type 2 diabetes mellitus had a complete remission of the disease and accordingly discontinued pharmacological therapy one month after surgery. Similarly, a remission of the disease was seen also for the five patients affected by insulin resistance.

The follow-up adherence reported in this study was equal to 97.5% (78). Two patients were lost during follow-up: one patient died of sepsis related to a gastric leak and the other left due to work-related hindrances.

### 3.2. Weight Loss

The mean and standard deviation of BMI at the different timepoints are reported in Table 2. Compared to the baseline value of 42, the mean value of BMI progressively decreases over time. At 1 month, the decrease is 9.5%, at 3 months it reaches 19.0%, at 6 months 28.6%, and at 12 months 35.7%.

**Figure 2:**
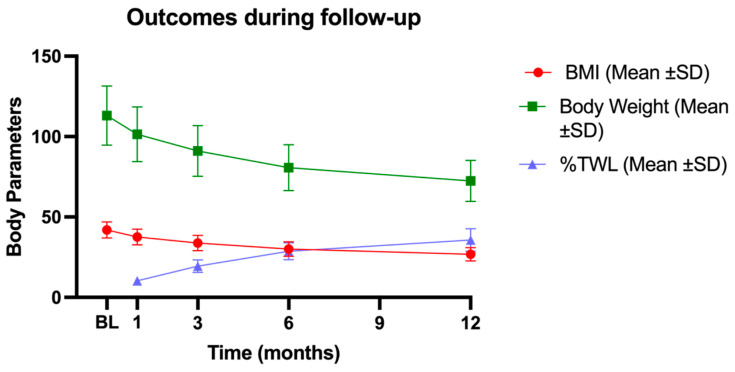
Body parameter trends during the follow-up. The figure shows the decrease in BMI and body weight and the progressive increase in % TWL. BL: baseline; 1M: one month; 3M: three months; 6M: six months; 12M: twelve months; BMI: body mass index; SD: standard deviation; %TWL: percentage of total weight loss.

Mean levels (±SD) of %TWL are reported in Table 2, and illustrated in Figure 2, in combination with other body parameters such as BMI and absolute bodyweight, to describe the weight loss outcomes.

### 3.3. Lipid Profiles

According to the repeated-measures (RM) one-way ANOVA test carried out, followed by Tukey’s multiple comparisons post hoc test, a significant difference in total cholesterol was highlighted across the timepoints (*p* < 0.0001). Tukey’s test showed a decreasing statistical significance trend between the baseline (BL) and the more distant timepoints. In particular, the decrease between BL and the one- and three-month timepoints was characterized by a higher level of statistical significance (*p* < 0.0001) when compared to the difference between BL and the six- and twelve-month timepoints (*p* = 0.0003 and *p* = 0.008, respectively).

The comparison between the three-, six-, and twelve-month timepoints showed no statistically significant differences, while a statistically significant increase was seen between the timepoint set at one month and the following ones.

HDL cholesterol levels were analyzed using an RM one-way ANOVA test, followed by Tukey’s multiple comparisons post hoc test, which highlighted a statistically significant difference between timepoints (*p* < 0.0001). In particular, the comparison between the BL and the 12-month timepoint showed a statistically significant increase (*p* < 0.0001). The increase in HDL cholesterol levels at the last timepoints was preceded by a significant decrease evidenced through the comparison between BL and the 1-month timepoint (*p* < 0.0001). After the second timepoint, an increasing trend is visible by comparing HDL cholesterol levels between BL and the 3- and 6-month timepoints, which showed no statistically significant differences. On the contrary, statistically significant differences were found when comparing the 3-, 6-, and 12-month timepoints, with a significant increase in the last timepoint when compared to the others (*p* < 0.0001).

LDL cholesterol levels were analyzed using an RM one-way ANOVA test, followed by Tukey’s multiple comparisons post-hoc test. In this case, only a significant decrease in the first timepoint compared to the others (*p* < 0.0001) was highlighted, suggesting a successive increase in the following. In fact, the difference between the BL and the 12-month timepoints was not statistically significant.

**Figure 3:**
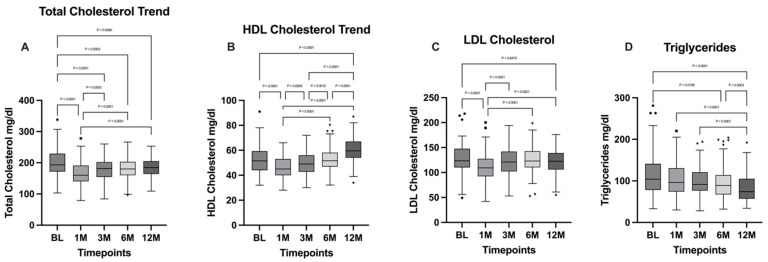
Comparison of (**A**) total cholesterol; (**B**) HDL cholesterol; (**C**) LDL cholesterol; and (**D**) triglycerides between the different timepoints. BL: baseline; 1M: one month; 3M: three months; 6M: six months; 12M: twelve months. The presence of markers (circles: BL, squares: 1M, upward triangles: 3M, downward triangles: 6M, rhombuses: 12M) indicates patients outside the range defined by the box plot, but not listed as outliers according to our statistical analyses.

The Friedman test with Dunn’s multiple comparisons post hoc test was performed to analyze the variance between the timepoints as regards triglycerides levels, showing a statistically significant difference between timepoints (*p* < 0.0001). Particularly, a general decreasing trend was evidenced, with no statistical significance between adjacent timepoints (BL vs. 1 month; BL vs. 3 months; 1 month vs. 3 months; 1 month vs. 6 months; 3 months vs. 6 months), except for the comparison between the 6-month and the 12-month timepoints, which showed a significant decrease in the latter (*p* = 0.0003). All the other timepoints, when compared to the 12-month one, show a statistically significant decrease (*p* < 0.0001). Total cholesterol, HDL cholesterol, LDL cholesterol, and triglycerides levels are illustrated in Figure 3.

### 3.4. Thyroid Hormones

The Friedman test conducted to analyze the TSH trend showed a statistically significant difference among the timepoints’ variances (*p* < 0.0001). The Dunn’s multiple comparison post hoc test highlighted a statistically significant decrease between the BL and the other timepoints (BL vs. 1M: *p* = 0.0039; BL vs. 3M: *p* < 0.0001; BL vs. 6M: *p* < 0.0001; BL vs. 12M: *p* = 0.0072), while no statistical significance was found in the comparisons between other timepoints.

As regards triiodothyronine (T3), the Friedman test carried out assessed the statistically significant difference between the timepoint variances (*p* < 0.0001), and the Dunn’s post hoc test evidenced a general decreasing trend, with a statistically significant decrease between the BL and the 12-month timepoint (*p* < 0.0001) and between the BL and the 6-month timepoint (*p* < 0.0001). In addition, a statistically significant decrease has been evidenced between the 3-month and 6-month timepoints (*p* = 0.0004).

The Friedman test conducted to analyze the differences between thyroxine (T4) levels showed no statistically significant results, and so did the Dunn’s multiple comparisons post hoc test, showing no statistically significant differences between individual timepoints. TSH, T3, and T4 levels are summarized in Table 3.

### 3.5. Liver Condition

Liver functionality markers such as AST and ALT were investigated using Friedman tests for both parameters, which showed a significant difference for both (*p* < 0.0001). Both AST and ALT levels were characterized by a similar trend: a significant increase at the one-month timepoint for AST (BL vs. 1M: *p* = 0.002) followed by a statistically significant decrease at the 12-month timepoint (BL vs. 12M: *p* < 0.0001); the increase at the one-month timepoint for ALT was not statistically significant (BL vs. 1M: *p* = 0.08), although the difference between the baseline and the 12-month timepoint was statistically significant (*p* < 0.0001). The difference between the 6-month and 12-month timepoints is not statistically significant for both AST and ALT.

**Figure 4:**
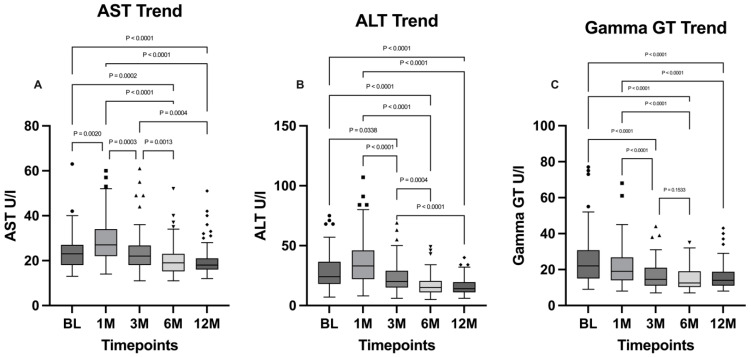
Comparison of (**A**) AST, (**B**) ALT, and (**C**) gamma GT levels between the timepoints. BL: baseline; 1M: one month; 3M: three months; 6M: six months; 12M: twelve months. The presence of markers (circles: BL, squares: 1M, upward triangles: 3M, downward triangles: 6M, rhombuses: 12M) indicates patients outside the range defined by the box plot, but not listed as outliers according to our statistical analyses.

Gamma GT (GGT) levels were analyzed using the Friedman test followed by Dunn’s multiple comparisons post hoc test. The general differences between the timepoints were statistically significant (*p* < 0.0001) and a general decreasing trend along the timepoints was evidenced. The comparison between GGT levels at baseline and at 3 months highlights a statistically significant decrease of this parameter (*p* < 0.0001), which is subsequently maintained in the following timepoints, in which no statistically significant differences were found. AST, ALT, and gamma GT levels are illustrated, respectively, in Figure 4.

### 3.6. Pancreatic Functionality and Glucose Levels

Pancreatic amylase levels were analyzed with the Friedman test, followed by Dunn’s multiple comparisons test. A significant difference between timepoints was evidenced (*p* < 0.0001) and the fluctuation trend of amylase is confirmed by the comparisons between the 1-month timepoint and the 3-month timepoint, in which a significant decrease has been evidenced (*p* < 0.0001), followed by a significant increase that emerged from the comparison between the 3- and 6-month timepoints (*p* = 0.0181). A further significant rise at 12 months was highlighted from the comparison with the 6-month timepoint (*p* < 0.0001).

As regards lipase, the Friedman test evidenced a significant difference between the timepoints (*p* < 0.0001) and the Dunn’s multiple comparisons test evidenced a significant raise at the one-month timepoint (BL vs. 1M: *p* < 0.0001), followed by a normalization of lipase to baseline levels.

The Friedman test carried out to analyze the glycated hemoglobin (HbA1c) ratio evidenced a statistically significant difference between timepoints (*p* < 0.0001). A general decreasing trend emerged from Dunn’s multiple comparisons test, particularly from the comparison between BL and the 12-month timepoint, in which a significant decrease was highlighted (*p* < 0.0001).

**Figure 5:**
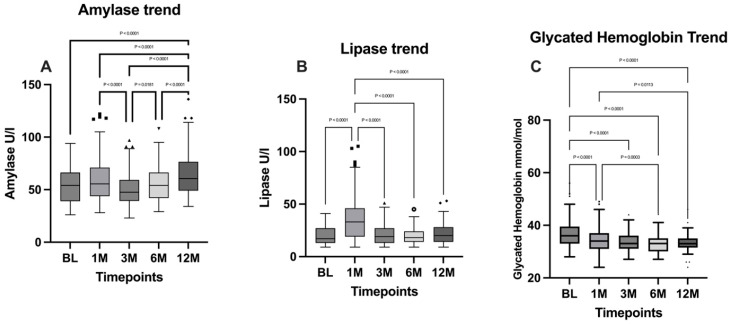
Comparison of (**A**) amylase levels; (**B**) lipase levels; and (**C**) glycated hemoglobin between the timepoints. BL: baseline; 1M: one month; 3M: three months; 6M: six months; 12M: twelve months. The presence of markers (circles: BL, squares: 1M, upward triangles: 3M, downward triangles: 6M, rhombuses: 12M) indicates patients outside the range defined by the box plot, but not listed as outliers according to our statistical analyses.

Amylase levels, lipase levels, and the glycated hemoglobin ratio are illustrated in Figure 5.

**Figure 6:**
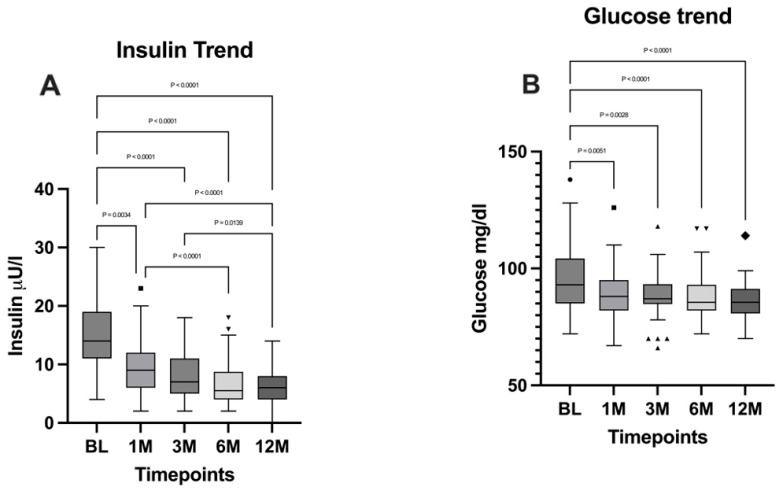
Comparison of (**A**) insulin levels and (**B**) glucose levels between the timepoints. BL: baseline; 1M: one month; 3M: three months; 6M: six months; 12M: twelve months. The presence of markers (circles: BL, squares: 1M, upward triangles: 3M, downward triangles: 6M, rhombuses: 12M) indicates patients outside the range defined by the box plot, but not listed as outliers according to our statistical analyses.

Both insulin and glucose levels were analyzed by performing the Friedman test, followed by Dunn’s multiple comparisons test, from which emerged a significant difference between the timepoints (*p* < 0.0001). A significant decrease emerged for both parameters when comparing the BL with the 12-month timepoint (*p* < 0.0001). Insulin and glucose levels are illustrated in Figure 6.

## 4. Discussion

Lack of adherence to follow-up protocols poses significant risks for post-bariatric patients, who require ongoing monitoring to ensure successful long-term outcomes [13,14].

Risk of complications such as nutritional deficiencies, surgical complications, and psychological distress can be mitigated by regular follow-up, which is crucial for identifying and managing these issues and preventing weight regain [14]. In addition, follow-up appointments provide an important opportunity to tailor treatment plans and dietary recommendations to the evolving needs of each patient. The literature highlights the importance of consistent follow-up to improve long-term outcomes after metabolic bariatric surgery [14]. Nevertheless, many studies show a gap in patient adherence to long-term follow-up, despite its proven benefits in short-term management [3].

Follow-up (up to three years) is widely regarded as essential to prevent weight regain and other postoperative complications. A systematic review by Reiber et al. confirms this, showing a significant association between adherence to follow-up and excess weight loss (EWL) within the first three years postoperatively [14], corroborating earlier findings by Kim et al. on the impact of follow-up after laparoscopic Roux-en-Y gastric bypass (LRYGB) [15].

This retrospective study suggests that MBS in combination with a high adherence to postoperative follow-up may be associated with significant improvements in various patient outcomes. Since 2022, a new structured follow-up protocol has been implemented in our care center, leading to the remarkable achievement of a 97.5% adherence rate during the 2022–2024 period. After one year, none of the patients in the cohort showed weight regain or insufficient weight loss; the mean BMI decreased from 42 to 27, demonstrating an efficient total weight loss.

Prolonged treatment interventions and continuous professional support can improve weight treatment outcomes [16]; moreover, it has been shown that patients who have complete trust in their healthcare practitioners are more likely to follow medical advice, thereby contributing to improved treatment outcomes [17].

Thanks to the implementation of a strict follow-up protocol, we were able to detect in our cohort multiple positive metabolic and biochemical changes due to MBS.

In accordance with the literature [18,19,20,21,22], total cholesterol and triglyceride levels decrease while HDL levels increase in our cohort of patients one year after surgery, reaching physiological levels. An interesting datum emerges from the LDL levels, which decrease with statistical significance one month after surgery; however, levels observed at the 12-month timepoint appear to return to the baseline; as far as we know this finding is not widely reported in the literature [21].

Another finding of our retrospective study is a significant decrease in thyroid-stimulating hormone (TSH) and triiodothyronine (fT3) levels. This result aligns with the existing literature, which suggests that weight loss leads to improved thyroid function [23,24,25]. The reduction in TSH and fT3 is likely due to the overall enhancement in metabolic health following metabolic bariatric surgery, which positively influences thyroid hormone regulation. In addition, we observed that free thyroxine (fT4) levels remained stable postoperatively.

A significant reduction in hepatic inflammation was observed, as evidenced by the statistically significant decrease in transaminase levels, particularly ALT, and gamma GT. The improvement in liver function underscores the essential role of weight loss in reducing hepatic inflammation and enhancing overall liver health, as reported in the literature [26,27,28].

Conversely, indicators of pancreatic function such as amylase and lipase levels showed an initial increase at one month postoperatively, likely reflecting the physiological trauma of surgery. At subsequent timepoints, pancreatic enzyme levels decreased, although they remained above baseline levels. This transient increase in pancreatic enzymes is consistent with the surgical stress response and their stabilization at elevated levels is reported in previous studies [29].

As expected, insulin, HbA1c, and blood glucose levels showed statistically significant and substantial reductions that were directly proportional to weight loss at 12 months postoperatively. In line with these data, all patients affected by type 2 diabetes mellitus or insulin resistance achieved complete remission and interrupted the pharmacological therapy after one month. These findings highlight the beneficial effects of metabolic bariatric surgery on glycemic control and overall metabolic health as reported in the literature [30,31,32].

## 5. Conclusions

In conclusion, this study provides insight into the biochemical changes that occur in post-bariatric patients who undergo a structured follow-up protocol. Our patient cohort experienced significant weight loss and demonstrated improved overall health outcomes without reporting any long-term complications. These findings contribute to understanding how MBS associated to a consistent follow-up may support enhanced patient outcomes by closely monitoring physiological changes and managing potential risks. While this study demonstrates the observational benefits of MBS, further research is needed to establish a direct link between follow-up adherence and clinical outcomes.

## Figures and Tables

**Table 1:** Patients’ comorbidities before metabolic bariatric surgery.

Comorbidity	N	Percentage
Cardiovascular diseases	23	28.75%
Hypothyroidism	12	15%
Type 2 diabetes mellitus	6	7.5%
GERD	5	6.25%
Insulin resistance	5	6.25%
Autoimmune thyroiditis	4	5%
Polycystic ovary syndrome	2	2.5%

N: number of patients affected by the comorbidity; GERD: gastroesophageal reflux disease.

**Table 2:** BMI and %TWL at the different timepoints of follow-up after metabolic bariatric surgery.

Timepoint	Mean BMI ± SD	Mean %TWL ± SD
Baseline	42 ± 5.05	-
1 month	38 ± 4.92	10.33 ± 2.58
3 months	34 ± 4.74	19.50 ± 3.92
6 months	30 ± 4.53	28.64 ± 5.35
12 months	27 ± 4.08	35.70 ± 7.05

BMI: body mass index; SD: standard deviation; %TWL: percentage of total weight loss.

**Table 3:** Summary of thyroid hormones levels at the different timepoints.

Variable	BL	1M	3M	6M	12M
TSH, μU/mL (median, IQR)	2.12 (1.43–2.84)	1.55 (1.05–2.56)	1.51 (1.09–2.09)	1.5 (1.06–2.16)	1.63 (1.23–2.33)
T3, pg/mL (median, IQR)	3.57 (3.26–3.95)	3.34 (3.1–3.63)	3.34 (3.14–3.57)	3.13 (2.89–3.35)	3.1 (2.78–3.41)
T4, ng/dL (median, IQR)	0.86 (0.76–0.97)	0.87 (0.78–0.99)	0.93 (0.80–1.02)	0.84 (0.75–0.95)	0.87 (0.72–1)

BL: baseline; 1M: one month; 3M: three months; 6M: six months; 12M: twelve months; IQR: interquartile range.

## Data Availability

The data supporting the reported results are not publicly available due to privacy and ethical restrictions.
